# The Equine Epizootic

**Published:** 1880-11

**Authors:** 


The Equine Epizootic That began in the New England States a short while since, has gradually extended itself to the Middle and Western States, and in some localities in the Southern States also. It appears to be of mild character, and though evidently depending upon the same cause as that of 1873, is not nearly so severe. Whilst the former visitation affected nearly all the horses in the States and Canada, and was frequently attended with fatal results; the latter is so mild in its character as scarcely to render horses laboring under it unfit for duty. A saline purgation, followed by nitre, if the horse continues feverish; and belladonna, when the symptoms call for its use, with flax seed, seem to be about all the remedies required.

